# lncRNA *1700009J07Rik* Impaired Male Fertility by Interfering with Sexual Behaviors in Mice

**DOI:** 10.3390/ijms26125801

**Published:** 2025-06-17

**Authors:** Hongyu Wang, Xiaojun Liu, Shijue Dong, Yang Zhou, Jingyan Yu, Meng Zou, Mengqian Ding, Aiwen Kang, Nanxi Ji, Xuhui Zeng, Xiaoning Zhang

**Affiliations:** 1 Institute of Reproductive Medicine, Medical School, Nantong University, Nantong 226019, China; 2431310067@stmail.ntu.edu.cn (H.W.); 1735718812@stmail.ntu.edu.cn (X.L.); 2113310047@stmail.ntu.edu.cn (S.D.); 2013310051@stmail.ntu.edu.cn (Y.Z.); 171701123@stmail.ntu.edu.cn (J.Y.); 2331310068@stmail.ntu.edu.cn (M.Z.); 2231310058@stmail.ntu.edu.cn (M.D.); aiwen.kang@shenghuisw.com (A.K.); 2231310055@stmail.ntu.edu.cn (N.J.); 2 Jiangsu Province Key Laboratory in University for Inflammation and Molecular Drug Target, Nantong University, Nantong 226019, China

**Keywords:** lncRNA, *1700009J07Rik*, spermatogenesis, male infertility, testosterone, sexual behavior

## Abstract

Long non-coding (lnc) RNAs exhibit tissue-specific expression characteristics and have been shown to be involved in the regulation of various biological processes. The testis is one of the organs with the most abundant lncRNAs. However, the functions of many testis-specific or -enriched lncRNAs in male fertility remain undisclosed. In this study, we screened lncRNA *1700009J07Rik* (*07Rik*) to investigate its roles in spermatogenesis and male fertility using knockout (KO) mice. We found that *07Rik* mainly acted as an intact lncRNA rather than a small protein, being highly expressed in various spermatogenic cells, which suggests its potential involvement in spermatogenesis. Unexpectedly, the deletion of *07Rik* did not impact spermatogenesis or sperm functions. Intriguingly, two-thirds of the male KO were infertile, which was ascribed to the lack of sexual behaviors rather than abnormalities in spermatogenesis or sperm functions. Further results reveal that, compared with wild-type mice, free testosterone content in serum was significantly reduced in the KO infertile (KO-I) mice, whereas it was remarkably elevated in the testes. Correspondingly, *Hsd3b2*, a key gene that promotes testosterone synthesis, was dramatically upregulated. *Cyp19a1* and *Cyp11b1,* which are responsible for testosterone metabolism, were downregulated in the testes. In addition, the expression of sex hormone-binding globulin was observably elevated in the testes of *07Rik* KO-I mice, which might partially explain the decrease in testosterone in the serum. These results suggest that disruptions in testosterone synthesis and metabolism might contribute to the loss of libido in *07Rik* KO-I mice. Our findings expand the understanding of lncRNA function and provide novel insights into the role of lncRNAs in male fertility, particularly in relation to hormonal turnover disorders that mediate sexual behavior defects.

## 1. Introduction

Long non-coding (lnc) RNAs are a type of RNA that is more than 200 bases long and does not traditionally encode proteins. Mounting evidence indicates that lncRNAs participate in various biological processes at multiple levels, including transcription, post-transcriptional processes, translation, and post-translational modifications, encompassing both genetic and epigenetic aspects across various species [1,2]. Notably, certain lncRNAs have been demonstrated to encode peptides derived from small open reading frames (ORFs) that play crucial roles in diverse physiological and pathological processes [3,4,5]. They have been found to be involved in cell proliferation, differentiation, cell cycle and apoptosis, either in the form of intact lncRNAs or small peptides. The dysfunction of lncRNAs is related to a variety of diseases, including cancers and male infertility [6,7,8]. In contrast to coding genes, lncRNAs exhibit more spatially and temporally specific expression patterns, lower expression levels and fewer vertebrate orthologs, and have undergone rapid evolution in sequences and functions across species [9,10]. Consequently, significant challenges persist in elucidating the roles and mechanisms of lncRNAs in specific tissues in distinct species. The most common strategy that explores the functions of lncRNAs involves employing knockout (KO) mouse models with the large fragment excision technology based on Clustered Regularly Interspaced Short Palindromic Repeats associated protein (CRISPR)/CRISPR-associated nuclease 9 (CRISPR-Cas9). This technology has clarified the functions of numerous lncRNAs; however, the specific functions of many specifically expressed or enriched lncRNAs in the testes remain largely unexplored.

The testes harbor the highest numbers and expression levels of lncRNAs, with many being testis-specific and exhibiting high expression levels in post-meiotic spermatids, suggesting their potential importance in male fertility [11,12]. Although numerous lncRNAs have been shown to be non-essential for spermatogenesis, some studies indicate their involvement in male subfertility or infertility through various mechanisms [13,14]. For instance, the overexpression of lncRNA *Gm2044* in spermatogonia has been linked to impaired spermatogenesis in some seminiferous tubules [15]. *ACVR2B-as1* interacts with ALDOA to regulate the self-renewal and apoptosis of human spermatogonial stem cells by modulating glycolysis activity [16]. *Teshl* is required for the expression of Y chromosome genes and maintaining a normal offspring sex ratio [17]. The polypeptides Kastor, Polluks and Tug1 encoded by those previously annotated as encoding lncRNAs are essential for spermatogenesis [18,19]. Additionally, mature sperm-borne lncRNAs not only affect sperm motility [20,21], but also participate in early embryonic development [22]. Hence, it is of great significance to continue exploring key molecules such as lncRNAs that regulate reproductive functions.

The spermatogenic process that occurs in the testes is essential for the production of male gametes. Normal spermatogenesis, spermiogenesis and sexual activity are crucial aspects of the male reproductive process, with the testes playing a pivotal role. In addition to the spermatogenic cells responsible for sperm production, somatic cells such as Leydig and Sertoli cells are vital for maintaining male reproductive function by providing regulatory factors, including hormones necessary for spermatogenesis and sexual desire [23]. The intricate mechanisms governing spermatogenesis and steroidogenesis in testicular cells remain largely unknown. Recent studies have interpreted that lncRNAs also play important roles in these cells by affecting steroidogenesis [24,25] and the blood–testis barrier [26]. Specifically, lncRNA *LOC102176306* and *XIST* function as sponges for miR-*1197-3p* and *miR-142-5p or RNA-145a-5p*, respectively, thereby targeting genes such as *Ppargc1a*, *StAR*, *3β-HSD and SIRT1* involved in the steroidogenesis pathway in Leydig cells under physiological or pharmacological stress conditions [27,28,29]. Steroidogenesis is crucial not only for spermatogenesis but also for regulating sexual behaviors. Disruptions of hormone synthesis and metabolism, particularly testosterone, in the testes can lead to impaired sexual behaviors, representing a significant cause of male infertility [30,31]. However, it is still unknown whether the highly expressed lncRNAs in the testes modulate hormone homeostasis driving sexual behaviors to mediate male fertility.

In this study, we screened lncRNAs that might have a regulatory effect on spermatogenesis and steroidogenesis from a large pool of highly-expressed testis-specific lncRNAs using gene co-expression network analysis via the MEM website (https://biit.cs.ut.ee/mem/, accessed on 1 June 2022) as research targets. Ultimately, we focused on *1700009J07Rik* (*07Rik*, Gene ID: 75188), because predictions have indicated that it might affect spermatid nucleus differentiation. Furthermore, *07Rik* is highly expressed in the testes, with expression levels gradually increasing along with the process of spermatogenesis. Next, we investigated its potential role in male reproductive processes, particularly in spermatogenesis, sperm function, testosterone synthesis and metabolism, and also in sexual behaviors utilizing Cas9 technology-generated KO mice. This research aims to uncover new functions of lncRNAs and their underlying mechanisms, and we hope to provide a novel perspective on the investigation of lncRNAs in male fertility and libido regulation.

## 2. Results

### 2.1. LncRNA 07Rik Was Highly Expressed in Mouse Testes

**Figure 1:**
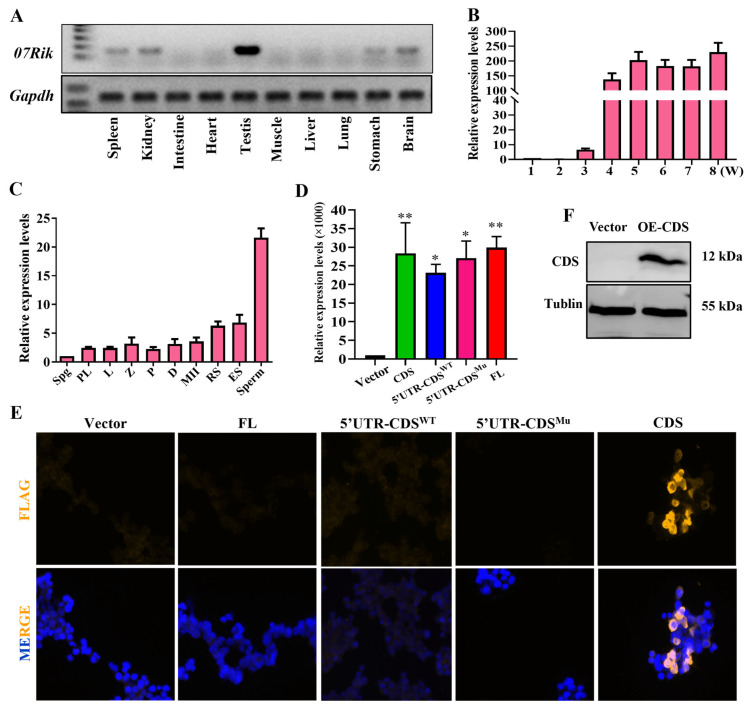
Expression profiles and characteristics of lncRNA *1700009J07Rik* (*07Rik*). (**A**) Expression levels of *07Rik* in various tissues. (**B**) *07Rik* expression levels in the testes at different development times and (**C**) in different spermatogenetic cells. (**D**) Overexpression levels of different *07Rik* transcripts in 293T cells. Spermatogenic cells were sorted using flow cytometry. Total RNA was extracted from each sample using a MiniBEST Universal RNA Extraction Kit and gene expression levels were detected by RT-PCR or qPCR. *Gapdh* was used as a loading control to normalize the gene expression levels in different samples. (**E**) Validation of protein-encoding abilities of different transcripts of *07Rik* by immunofluorescence (magnification, 200×). (**F**) The expression levels of *07Rik* CDS encoded a small protein in 293T cell lines. Different *07Rik* sequences with the flag tag were constructed into a Eukaryotic expression vector and then transfected into 293T cells. Immunofluorescence staining and Western blot with a Flag antibody was used to verify the expression of the presumed fusion protein. The tubulin protein was used as a loading control. All data are means ± SEM (*n* = 3). *p* values are calculated using the Tukey–Kramer test. * *p* < 0.05, ** *p* < 0.01, compared with the vector control group. Spg, spermatogonia; PL, preleptotene spermatocytes; L, leptotene spermatocytes; Z, zygotene spermatocytes; P, pachytene spermatocytes; D, diplotene spermatocytes; MII, meiosis II spermatocytes; RS, round spermatids; ES, elongated spermatids; Sperm, spermatozoa; OE, Overexpression; FL, full length.

Firstly, the expression profiles of *07Rik* across various tissues and developmental stages of testes and spermatogenic cells in mice were investigated. The results indicate that it was highly expressed in the testes, with lower levels observed in the spleen, kidney, and brain (Figure 1A). During the initial spermatogenic wave, *07Rik* expression levels were significantly increased in the testes at three weeks of postnatal development (Figure 1B). Notably, during the continuous spermatogenesis stage, *07Rik* was expressed in all spermatogenic cell types, with a pronounced enrichment in haploid cells (Figure 1C, Supplemental Figure S1). Interestingly, it was also presented in Leydig and Sertoli cells (Supplemental Figure S1B). These results suggest that *07Rik* might play a crucial role in male fertility. Next, we aimed to determine whether *07Rik* functions as a non-coding RNA in vivo. The Coding Potential Assessment Tool (http://lilab.research.bcm.edu/index.php, accessed on 8 November 2020) and ORF Finder (https://www.ncbi.nlm.nih.gov/orffinder/, accessed on 1 November 2020) showed that *07Rik* could encode a small protein consisting of 86 amino acids (Supplemental Figure S2). To verify this, we used an in vitro expression system with a eukaryotic expression plasmid (Supplemental Figure S3) and mass spectrometry to investigate the potential proteins derived from *07Rik’* ORF. Our findings show the successful transfection and transcription of the coding sequence (CDS), 5′UTR-CDS^WT^, 5′UTR-CDS^Mu^, and full-length sequences of *07Rik* in 293T cells (Figure 1D). However, only the cells transfected with the CDS expressed the predicted small protein (Figure 1E,F). Mass spectrometry did not detect this protein in the small-molecular-weight protein fractions of the testes (Supplemental Table S1). These results suggest that *07Rik* may function as an lncRNA, with regulatory effects on male fertility in vivo.

### 2.2. Deletion of 07Rik Did Not Affect Spermatogenesis

**Figure 2:**
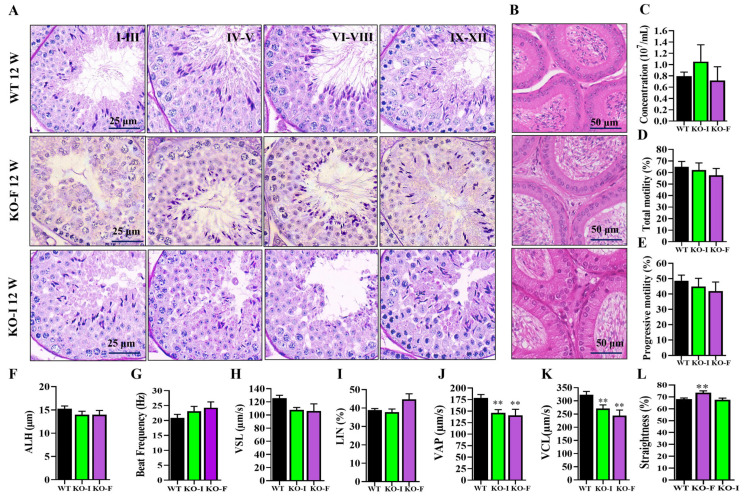
The effect of *1700009J07Rik* (*07Rik*) deletion on the development and morphology of testis and epididymis, and sperm parameters. (**A**) Development and histomorphology of the testis in adult knockout (KO) and wild-type (WT) male mice. (**B**) Development and morphology of the epididymis. Histopathological analyses of the testis and epididymis were performed by periodic acid-Schiff + hematoxylin and hematoxylin + eosin staining, respectively. Representative images from stages I–III, IV–V, VI–VIII and IX–IIX of the spermatogenesis progress and caput epididymis are shown. (**C**–**L**) Evaluation of sperm motility parameters in KO and WT mice. Sperm were collected from the caudal epididymis and we analyzed sperm count and motility on a computer assisted sperm analysis system. (**C**) Concentration, (**D**) total motility, (**E**) progressive motility, (**F**) ALH (amplitude of lateral head displacement), (**G**) beat frequency, (**H**) VAP (average path velocity), (**I**) VSL (straight-line velocity), (**J**) VCL (curvilinear velocity), (**K**) LIN (linearity), and (**L**) straightness were recorded, respectively. All data are means ± SEM (*n* > 3). *p* values were calculated using multiple comparison with Tukey–Kramer test. ** *p* < 0.01, compared with the group of WT.

Next, we conducted a detailed analysis of the effects of *07Rik* deletion on spermatogenesis, sperm count, and motility in male mice. As illustrated in Figure 2A, spermatogenesis in both *07Rik* knockout mice with infertility (KO-I) and knockout mice with fertility (KO-F) was largely unaffected. Similarly, no significant differences were observed in sperm number and morphology in the epididymis (Figure 2B). Interestingly, most sperm motility parameters measured here, including sperm count, total and progressive motility, amplitude of lateral head displacement (ALH), beat frequency (Figure 2C–G), straight-line velocity (VSL) (Figure 2H), and linearity (LIN) (Figure 2I), were comparable to those of wild-type (WT) mice. However, there was a slight decrease in average path velocity (VAP) and curvilinear velocity (VCL) (Figure 2J,K), while straightness showed a minor increase (Figure 2L). These findings suggest that *07Rik* may play a subtle role in spermatogenesis and sperm motility, which may not be sufficient to cause male infertility.

### 2.3. Deletion of 07Rik Impaired Male Fertility by the Loss of Mating Behavior

**Figure 3:**
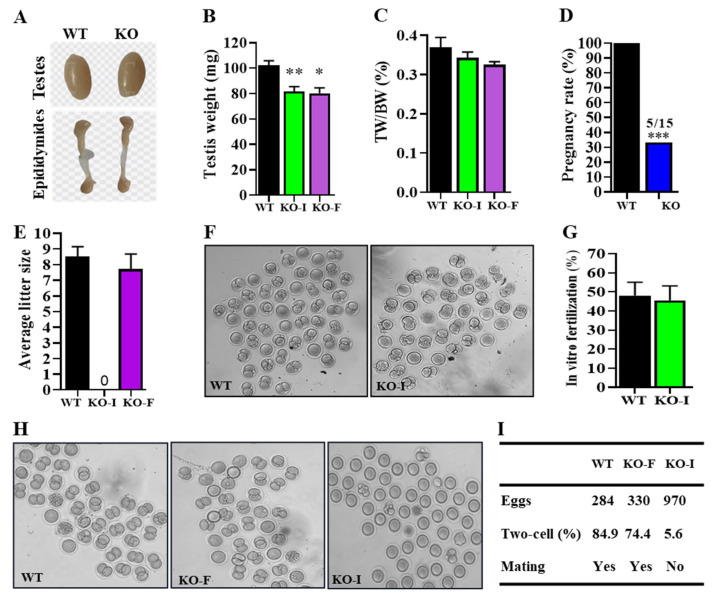
The knockout (KO) of *1700009J07Rik* (*07Rik*) did not affect sperm function but impaired male fertility by obstructing sexual behaviors. (**A**) External morphological comparison of the testis and epididymis in KO and wild-type (WT) mice. (**B**) Contrast of testes weight. (**C**) Ratio of testicular weight to body weight. (**D**) Pregnancy and (**E**) litter size between WT and KO mice. (**F**) The representative pictures of two-cell embryos developed from WT and KO mice with infertility (KO-I) sperm by in vitro fertilization (magnification, 100×). (**G**) Statistical analysis of the two-cell formation rate from (**F**). (**H**) Analysis of in vivo fertilization and mating behaviors in the *07Rik* KO male mice. Representative pictures of two-cell embryos developed from WT, KO mice with fertility (KO-F) and KO-I sperm (magnification, 100×). (**I**) The formation rate of two-cell embryos and mating behavior recording. Videos of sexual acts of male mice were recorded over a long period of time (12 h) after being caged with 2 female mice in estrus with hormone stimulation. The ampulla of the fallopian tube was dissected the next day and the development of two-cell embryos was statistically analyzed. All data are means ± SEM (*n* = 10–15). *p* values were calculated using a non-paired “Student-t” test or multiple comparison with Tukey–Kramer test. * *p* < 0.05, ** *p* < 0.01, *** *p* < 0.001, compared with the WT group.

Further phenotypic analysis showed no significant differences in the morphology and size of the testes and epididymis between KO and WT mice (Figure 3A). Despite the reduction in testicular weight, there was no significant change in the ratio of testis-to-body weight (Figure 3B,C). As previously mentioned, since *07Rik* KO did not disturb spermatogenesis, we sought to determine whether *07Rik* deletion affects male fertility by impairing sperm motility or functions. Amazingly, the fertility test results indicate that two-thirds of male KO mice were completely infertile (Figure 3D), while the number of pups per litter amongst the KO-F mice showed no substantial difference compared to WT mice (Figure 3E). The two-cell embryo rate from KO-F male mouse sperm was equivalent to that of WT mice in both in vitro (Figure 3F,G) and in vivo (Figure 3H,I) fertilization experiments. However, for KO-I male mice, few normal two-cell embryos were successfully developed via in vivo fertilization in female partners (Figure 3H,I).

**Figure 4:**
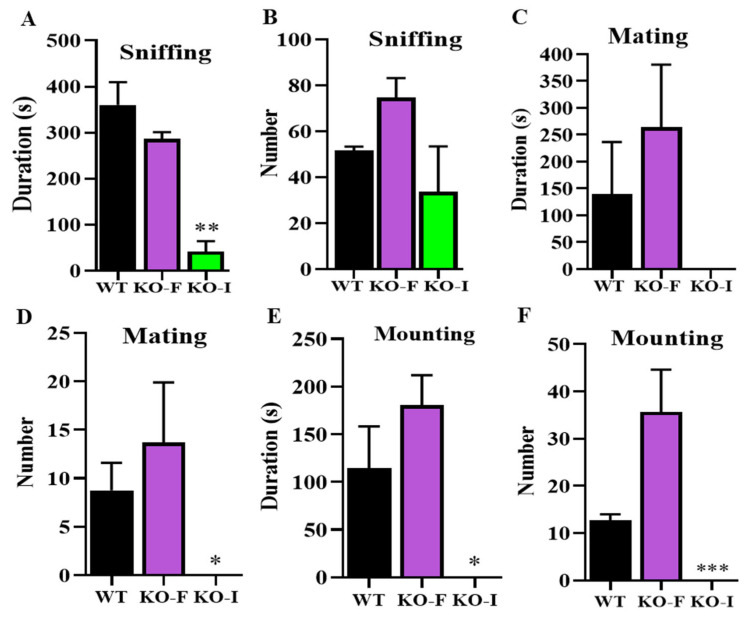
The evaluation of sexual behaviors in the *1700009J07Rik* (*07Rik*) knockout (KO) mice. (**A**,**B**) Duration and number of sniffing behaviors. (**C**,**D**) Duration and number of mating behaviors. (**E**,**F**) Duration and number of mount behaviors. The experimental male mice were caged 1:2 with ICR female mice in estrus stimulated by artificial hormones PMSG and HCG in advance. Sexual behaviors were video-recorded and the above parameters were analyzed in detail for each male mice after a 4 h caging period. All data are means ± SEM (*n* = 4). *p* values were calculated via multiple comparison with Tukey–Kramer test. * *p* < 0.05, ** *p* < 0.01, *** *p* < 0.001, compared with the WT group. PMSG, pregnant mare serum gonadotropin; HCG, human chorionic gonadotropin.

Hence, we speculated that KO-I male mice might not engage in mating at all, and subsequent long-term video surveillance confirmed our hypothesis (Figure 4). After assigning two female mice in estrus, stimulated by artificial hormones, to KO-F and KO-I male mice, we observed that the sexual desire of *07Rik* KO-I male mice was significantly reduced, resulting in no mating behaviors. The results of the sexual behavior analysis demonstrate that *07Rik* KO-I mice underwent a significant loss or decrease in sniffing (Figure 4A,B), mating (Figure 4C,D) and mounting (Figure 4E,F) responses toward their female mates (Supplemental Videos S1 and S2).

### 2.4. Abnormalities in Testosterone Synthesis and Metabolism Might Be Responsible for the Reduced Libido in 07Rik KO-I Mice

**Figure 5:**
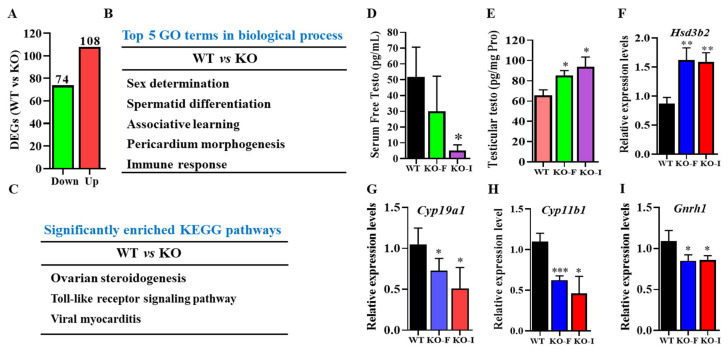
*1700009J07Rik* (*07Rik*) excision indirectly disturbed testosterone synthesis and metabolism. After RNA isolation, RNA-sequencing was performed to detect the differentially expressed genes (DEGs) in *07Rik* knockout (KO) mouse testes. The functions and pathways affected by DEGs were analyzed by GO and KEGG enrichment, respectively. (**A**) The number of DEGs in the testes after *07Rik* cancellation. (**B**) Biological processes significantly influenced by DEGs. (**C**) KEGG pathways markedly disturbed by DEGs. The contents of free testosterone in the serum (**D**) and testes (**E**). Free testosterone concentration was detected using a commercial Elisa kit. (**F**) *Hsd3b2*, (**G**) *Cyp19a1*, (**H**) *Cyp11b1*, and (**I**) *Gnrh1* transcript expression levels in KO and wild-type (WT) male mouse testes. Gene expressions were determined by qPCR assay. *Gapdh* was used as a loading control to normalize the gene expression levels. All data are means ± SEM (*n* = 3–10). *p* values were calculated using multiple comparison with Tukey–Kramer test. * *p* < 0.05, ** *p* < 0.01, *** *p* < 0.001, compared with the WT group. Testo: testosterone.

To elucidate the mechanisms underlying the reduced libido and absence of mating in *07Rik* KO-I male mice, RNA-seq was utilized to identify differentially expressed genes (DEGs) in mouse testes. As depicted in Figure 5A and Supplemental Table S2, 74 genes were down-regulated and 108 were up-regulated in *07Rik* KO mouse testes compared to WT mice. Notably, the functions of one-third of these dysregulated genes have not been reported previously. Next, Gene Ontology (GO) and Kyoto Encyclopedia of Genes and Genomes (KEGG) analyses revealed that the DEGs primarily impacted sex determination, spermatid differentiation, steroidogenesis, and toll-like receptor signaling pathways (Figure 5B,C). Considering free testosterone’s critical role in the regulation of sexual behaviors, serum and testicular testosterone levels were measured. Compared to WT mice, KO-I mice exhibited significantly reduced serum testosterone levels but increased testicular testosterone levels (Figure 5D,E). Correspondingly, the expression of the *Hsd3b2* gene, which enhances testosterone synthesis, was markedly upregulated, while the genes *Cyp19a1* and *Cyp11b1*, which are involved in testosterone metabolism, were downregulated (Figure 5F–H). Additionally, *Gnrh1*, an upstream regulator of testosterone synthesis, was down-regulated in *07Rik*-deficient mice (Figure 5I). Collectively, these findings suggest that disruptions in testosterone synthesis and metabolic pathways in the testes may have significantly contributed to the decreased libido in KO-I male mice.

## 3. Discussion

The functions of coding genes in the testes have been extensively studied at the genetic and epigenetic levels, yet the regulatory mechanisms governing physiological and pathological processes in male reproduction remain largely unexplored. Therefore, a comprehensive investigation into the roles of numerous non-coding genes, including lncRNAs, holds promise for advancing the diagnosis and treatment of male infertility. Accumulating studies have identified several testis-related lncRNAs and demonstrated their significant impacts on male fertility in in vivo models [17,32]. However, the functions of the majority of lncRNAs in mammalian testes remain unclear [14,33]. In this research, we examined the lncRNA *07Rik*, which exhibited expression levels comparable to the housekeeping gene *Gapdh*, as indicated by the cycle threshold value in the qPCR assay, to assess its effects on mouse spermatogenesis and fertility. Our study demonstrates that *07Rik* is dispensable in spermatogenesis but plays a crucial role in indirectly regulating testosterone-mediated sexual behaviors. The deletion of *07Rik* resulted in reduced libido and impaired fertility in male mice.

Our data reveal that *07Rik* exhibited high enrichment (FPKM = 95–209) in mouse testes. Following the development of spermatogenic cells through the haploid process during initial spermatogenesis, its expression markedly increased and remained elevated until the mature sperm stage. This expression profile resembled that of numerous other testis-specific lncRNAs [34,35], indicating that *07Rik*, akin to spermatogenic cell-specific coding genes [36], likely played a predominant role in regulating spermiogenesis, maturation, or sperm functions. The notable expression of *07Rik* in mature sperm suggests its potential transmission to the fertilized egg post-fertilization, potentially facilitating the transgenerational inheritance of epigenetic information. Subsequent data reveal the expression of *07Rik* in spermatogonia, spermatocytes, Leydig cells, and Sertoli cells, implying its broader involvement in male reproduction, which warrants further exploration.

Traditionally, lncRNAs exert sophisticated functions primarily through their transcripts. Recent studies, however, have revealed that lncRNAs can also contain ORFs capable of encoding small proteins [37,38]. Two notable examples in male reproduction are Kastor and Polluks, proteins encoded by lncRNA ORFs, which integrated into the outer mitochondrial membrane and interacted with the voltage-dependent anion channel, thus impairing male fertility [18]. Additionally, the lncRNA locus *Tug1*, crucial for fertility, functioned not only through RNA-based cis- or trans-mechanisms, but also via a small protein encoded by an evolutionarily conserved ORF with a non-canonical start codon [19]. Thus, the primary question is whether *07Rik* qualifies as a classical lncRNA. Bioinformatics analysis suggested that *07Rik* might encode a small protein; however, this prediction was not supported by in vitro and in vivo experimental evidence in our study. Despite its coding potential, the actual in vivo situation may differ, as lncRNAs also possess 5′ regulatory elements, and the predicted protein has not been detected via mass spectrometry. It is possible that the expression level of the small protein encoded by the *07Rik* ORF is too low or unstable to be detected. Overall, *07Rik* likely functions predominantly through its transcript.

Next, the analysis of testicular development revealed that *07Rik* KO mice had lighter testes compared to WT mice; however, the testes-to-body weight ratio was unchanged, indicating a potential impact on overall growth. Fertility tests showed that only one-third of the KO mice were fully fertile. Interestingly, PAS staining revealed that spermatogenesis was unaffected in both KO-F and KO-I mice, which is consistent with recent findings suggesting that many lncRNAs are non-essential for spermatogenesis in mice [35,39,40,41]. In addition, most sperm motility parameters were unaffected, providing additional support for the notion that the involvement of lncRNAs in testicular function may be non-critical or context-dependent. Subsequent fertilization tests demonstrated that sperm from both KO-I and KO-F mice retained an equivalent fertilization capacity compared with WT mice. Sexual behaviors analysis confirmed these findings, revealing a significant reduction in absence of sniffing, mounting, and mating duration, as well as a decrease in the amount of mounting and mating in KO-I male mice. Interestingly, we found that only two-thirds of the KO male mice were completely infertile due to the absence of sexual behaviors, while the remaining mice were normally fertile. In fact, we are not clear about the reason for this phenotypic heterogeneity phenomenon in our study. A possible explanation is that the deletion of *07Rik* leads to significant individual differences in hormone levels including testosterone and the expression of steroidogenesis genes related to sexual behaviors. Only mice with those differences reaching a certain threshold lost their sexual desire. Specific and clear causes and underlying mechanisms still require further investigation in the future. Nevertheless, our findings demonstrate that the deficient copulatory behaviors observed in *07Rik* KO-I mice are responsible for their complete infertility, rather than abnormal spermatogenesis or impaired sperm functions.

Hormone synthesis disorders that lead to reduced sexual activity are significant contributors to male fertility issues. Previous research has indicated that the deletion of testis-specific genes could result in impaired mounting behaviors due to abnormal steroidogenesis [42]. LncRNAs such as *CIRBIL* and *Start*, expressed in spermatogenic and Leydig cells, similarly impacted testosterone synthesis [24,25], which indicates that lncRNAs might contribute to male reproductive progress via steroidogenesis-mediated sexual activity. Our study involved a functional enrichment analysis of DEGs using RNA sequencing data, revealing that the deletion of *07Rik* significantly disrupted pathways related to sex determination, steroidogenesis, spermatid differentiation, associative learning, and others. Subsequent findings have confirmed dysregulated hormone regulation, particularly in testosterone synthesis and metabolism, which could significantly contribute to decreased libido. In the present study, the free testosterone content was remarkably lowered in the serum, but what is interesting is that it was higher in the testes of KO mice. We have hypothesized that the reduced testosterone levels in the bloodstream might stimulate the testes to increase testosterone production through potential feedback mechanisms. However, the testosterone produced by the testes may not be effectively transported or diffused into the bloodstream. A fact that cannot be ignored is that other factors might also influence testosterone turnover. The adrenal glands and liver also play crucial roles in testosterone synthesis and metabolism, respectively [43,44]. Dysfunctions in these organs can result in abnormal testosterone levels. However, given that *07Rik* is highly enriched in the testes, our research only focused on its testicular function. Future studies should explore the expressions and functions of *07Rik* in other tissues to gain a comprehensive understanding of its roles beyond the testes.

We further confirmed our conclusion by investigating the expressions of genes related to testosterone metabolism in the testes. 3β-HSD regulates testosterone synthesis, while Cyp19a1 and Cyp11b1 are primarily responsible for testosterone metabolism [45]. GnRH1 plays a dual role in endocrine regulation in the nervous system and the local modulation of testosterone synthesis in vertebrate testes [46,47]. Following the deletion of *07Rik* in mice, our findings show an upregulation of *3β-HSD*, accompanied by a downregulation of *Cyp19a1*, *Cyp11b1*, and *Gnrh1*. This effect is likely attributable to the expressions of *07Rik* in Leydig cells. Additionally, the increased expression of GATA4 in Sertoli cells, as observed in KO-I mouse testes, might also contribute to these changes (Supplemental Figure S4) [48]. The observed increase in testicular testosterone content does not account for the reduced serum testosterone levels in KO mice compared to WT mice. To explore this discrepancy, we examined sex hormone-binding globulin (SHBG), which influences free testosterone levels. Our results show a significant increase in SHBG protein levels in *07Rik* KO-I mouse testes (Supplemental Figure S4), suggesting that elevated SHBG may impede the release and transport of free testosterone into the bloodstream. Therefore, we have proposed that the loss of sexual activity caused by the lowered free testosterone was indirectly mediated by *07Rik* deletion, and testosterone supplementation might help remedy this defect in the future [49,50]. Nevertheless, it is still unknown how *07Rik* regulated the expression of hormone synthesis-related genes in the testes.

Sexual behaviors are predominantly regulated by the neuroendocrine system [51], suggesting a potential involvement of *07Rik* expression in the brain in this process. Notably, low levels of *07Rik* expression were observed across various brain regions (Supplemental Figure S5). While its expression in spermatogenic cells was much higher compared to somatic cells such as Leydig, Sertoli, and brain cells, its impacts on spermatogenesis and sperm function appeared minimal. This suggests the imperceptible regulatory role of *07Rik* in the reproductive process under normal physiological conditions, with potential regulatory functions emerging under specific adverse stresses, akin to other lncRNA genes [52,53]. Another possible explanation was that other genes compensated for the effects of the *07Rik* knockout on male fertility [54]. Furthermore, the high expression of *07Rik* in germ cells suggests a possible transfer to somatic cells via exosomes in order to modulate testosterone synthesis and turnover, facilitated by the ability of exosomes or their cargo from germ cells to traverse the blood–testis barrier, thereby promoting intercompartmental communication between seminiferous tubules and the interstitium [55]. Nevertheless, these hypotheses warrant validation with reference to robust experimental evidence in future studies.

In summary, our study is the first to identify the testis-enriched lncRNA *07Rik*, which, while not essential for spermatogenesis, plays a fine-tuning role in sperm motility and significantly influences libido by indirectly affecting testosterone turnover in mouse testes. This conclusion is supported by observations of diminished sexual behaviors, decreased serum free testosterone levels, increased testosterone levels in the testes, and the abnormal expression of genes involved in steroidogenesis pathways. This research narrows down the exploration of lncRNA’s roles in spermatogenesis, and offers insights into the impact of lncRNAs on male reproductive processes, particularly in hormonal disturbances that affect sexual behaviors.

## 4. Materials and Methods

### 4.1. Animals

The *07Rik* KO mouse was constructed with CRISPR-Cas9 technology by the Caygen Biosciences Company (Guangzhou, China). As depicted in Supplemental Figure S6A, a pair of gRNAs was designed to target a 3274 bp segment of genomic DNA at the *07Rik* locus on chromosome 10. Briefly, Cas9 mRNA and a designed pair of gRNAs (gRNA1: 3′-GCAGTGCCACAGACGGTGCGGGG-5′ and gRNA2: 3′-CTGAAGGTCGGTTGCATCCAGGG-5′) were pooled and co-injected into the zygotes from C57BL/6 mice, and KO mice were generated after two cell embryos were transferred into the female pseudo-pregnant ICR mice. Founder 1 KO mice were confirmed by PCR-based genotyping and Sanger sequencing. Animal genotyping was performed by PCR with genomic DNA isolated from tail clips of mice. The WT allele generated a band at 637 bp, while the KO allele was at 780 bp (Supplemental Figure S6B,C). Finally, the qPCR analysis of testis tissue confirmed the absence of *07Rik* transcript expression, verifying the successful KO of this lncRNA (Supplemental Figure S6D). The primer sequences are listed in Supplemental Table S3.

The mice were raised in a specific pathogen-free (SPF) barrier (SYXK (Su) 2025–0039, 21 May 2025) with controlled lighting (12 h light–dark cycle) and temperature (24–28 °C), and provided with standard food and water. All experimental methods were reviewed and approved by the Ethics Committee of the Animal Center of Nantong University. For the fertility test, 8-week-old sexually mature male KO mice and WT female mice were caged 1:2 for three months. They were then paired and checked again by caging with the other two WT female mice for an additional three months. The numbers of newborn pups per litter from each female were recorded and analyzed.

### 4.2. Hematoxylin–Eosin (HE) and Periodic Acid-Schiff (PAS) Staining

The testes were dissected immediately after the mouse was euthanized and then fixed in testicular tissue fixative fluid (G1121, Servicebio, Wuhan, China) for 24 h. Subsequently, the fixed tissues were stored in 70% ethanol until use. Next, the dehydration of the testicular tissue was accomplished by employing a series of alcohol solutions, followed by tissue transparency tests with xylene. The sample was then embedded in paraffin and sectioned into 5 µm slices using a microtome (PM2235 cwEU, Leica, Shanghai, China). Finally, these sections were mounted onto glass slides.

For HE staining, the sections were subjected to dewaxing with xylene and hydration using a gradually decreasing concentration of ethanol. After washing with water for 2 min, the sections were stained with hematoxylin for 5 min, followed by another wash with water for 10 min. Subsequently, the sections were stained with eosin solution for 2 min. PAS staining was conducted according to the instructions provided by the manufacturers (Solarbio, Beijing, China). The dewaxed and hydrated sections were treated with periodic acid solution for 10 min, followed by a water wash for 5 min. Subsequently, the slides were stained with Schiff reagent and hematoxylin. Finally, all sections were dehydrated, made transparent, and mounted using neutral resin. The slides were captured with a Leica AperioVERSA (Leica, Buffalo Grove, IL, USA).

### 4.3. Analysis of Sperm Counts and Motility

After the mice were euthanized, the clean cauda epididymis was removed and poured into a dish with 3 mL HS (Hepes solution, 135 mM NaCl, 5 mM KCl, 1 mM MgSO_4_, 2 mM CaCl_2_, 20 mM Hepes, 5 mM glucose, 10 mM lactic acid and 1 mM Na-pyruvate at pH 7.4 with NaOH) at 37 °C for 10–15 min. After the sperm had fully swam out, it was mixed and pipetted at 20 μL onto a designated chamber (Songjin Tianlun, Nanning, China) for the assessment of parameters via computer-assisted sperm motility analysis (CASA, Hamilton Thorne, Beverly, MA, USA). Each sample contained at least 1000 sperm from 6 regions, and we recorded parameters including concentrations, total motility, progressive, VAP, VSL, VCL, ALH, beat frequency, straightness, LIN, elongation and area.

### 4.4. In Vitro and In Vivo Fertilization

The female ICR mice were injected with pregnant mare serum gonadotropin (PMSG, 10 IU/L) followed by human chorionic gonadotropin (HCG, 10 IU/L) for superovulation after 48 h. The next day, MII oocytes isolated from female mice were prepared for the in vitro fertilization experiment. The sperm of WT and KO mice were collected from cauda epididymides and placed into human tubal fluid (HTF) (93.8 mM NaCl, 171.6 μM MgSO_4_, 4.69 mM KCl, 370 μM KH_2_PO_4_, 2.04 mM CaCl_2_, 21.40 mM Lactic acid, 2.78 mM Glucose, 21 mM Hepes, 4 mM NaHCO_3_, adjust PH to 7.4 with NaOH) for 1 h to achieve capacitation. Next, the capacitated sperm were covered over the surface of egg cells located in HTF, which had been predigested from cumulus cells with hyaluronidase. After incubation for 4–6 h, the zygote was washed as clean as possible in HTF, and subsequently transferred into KSOM medium (Aibei, Nanjing, China) for further development at 37 °C in a 5% CO_2_ incubator. The ratio of two-cell embryos was counted after 24 h. For in vivo fertilization, 2 female mice in estrus were administrated artificial hormones PMSG and HCG in advance, as described above, and were caged with male mice overnight. The next morning, the female mice were brought back to retrieve the fertilized eggs from the ampulla of the fallopian tube by digesting the cumulus cells with hyaluronidase. The ratios two-cell embryos were collected and analyzed. At the same time, the sexual behaviors of mice were recorded as follows.

### 4.5. Sexual Behaviors Recording and Analysis

Over twenty male mice were evaluated for their sexual behaviors at ages ranging from 3 to 6 months. Each mouse was tested twice with two different female spouses, with each test spaced at least one week apart, and their behaviors were videoed overnight for subsequent analysis. The definitions and recording criteria for male behavioral parameters were described in a previous study [56]. Briefly, the experimental male mice were housed in a 1:2 ratio with ICR female mice, and the female mice had been administered PMSG and HCG in advance to induce estrus to ensnare high sexual receptivity. For each male, the indicators of sexual behavior were analyzed in detail after a 4 h caging period. The recorded behavioral indexes were as follows: number and duration of sniffing episodes, number and duration of mating behaviors and number and duration of mounting behaviors.

### 4.6. Reverse Transcription and Quantitative Polymerase Chain Reaction (RT- and qPCR)

Total RNA was isolated from the testes, different brain region tissues and 293T cells, respectively, using a MiniBEST Universal RNA Extraction kit (TaKaRa, Tokyo, Japan). Then, the cDNAs were synthesized using a kit of HiScript III RT SuperMix for RT-PCR or qPCR (Vazyme, Nanjing, China). Next, we incorporated the PCR master mix containing ChamQ Universal SYBR into a LightCycler96 real-time PCR system (Roche, Mannheim, Germany) with the following reaction conditions: 98 °C for 1 min, 40 cycles of 98 °C for 10 s, 60 °C for 20 s and 72 °C for 20 s. For RT-PCR, after PCR amplification, the PCR products were analyzed on 2% agarose gels and visualized by ethidium bromide staining. *β-actin* or *Gapdh* was used as an internal control for standardizing the target gene expression. The RT-PCR and qPCR primers are listed in Supplemental Table S3.

### 4.7. RNA-Sequencing and Bioinformatic Analysis

The total RNA qualified by Agilent 2100/2200 Bioanalyzer (Agilent Technologies, Palo Alto, CA, USA) and NanoDrop (Thermo Fisher Scientific, Waltham, MA, USA) was used for RNA sequencing. Here, 1 μg total RNA was utilized for the following library preparation according to the manufacturer’s protocol. Sequencing was conducted using a novaseq 6000 (Illumina, San Diego, CA, USA) with PE150 according to the manufacturer’s instructions developed by GENEWIZ (Suzhou, China). Raw data were processed by Cutadapt (Version: v1.9.1) to derive high-quality clean data, which were further aligned to the mouse reference genome via software Hisat2 (v2.0.1). Gene expression levels were estimated using HTSeq (v0.6.1). Next, gene differential expression was analyzed by the DESeq2 Bioconductor package with a model based on the negative binomial distribution. GOSeq (v1.34.1) was used, identifying GO terms that annotate a list of enriched genes with a significant padj ≤ 0.05. We used in-house scripts to enrich the significantly differentially expressed genes in the KEGG pathways.

### 4.8. Determination of Free Testosterone Content

After the application of anesthesia, blood samples were collected from the eyes of mice and placed in centrifuge tubes, which were then kept at room temperature for 2 h. Then, the samples were centrifuged at 3000× *g* for 10 min, and the upper serum was collected and stored at −80 °C until use. Meanwhile, the isolated testes were thoroughly lysed in the phosphate buffer saline solution using a homogenizer and combined ultrasonication at 4 °C to fully release the testosterone within cells. The dialysate containing free testosterone was obtained from the serum or testicular homogenate supernatant using ultrafiltration with a molecular weight cut off of 10 kDa at 5000× *g* for 10 min, and then the supernatant was used for the detection of the free testosterone content. Free testosterone levels were measured with a Mouse Free Testosterone (Testo) ELISA Kit (D751047, Sangon biotech, Shanghai, China) according to the product manufacturer’s instructions. In order to achieve specific and accurate determination of free testosterone, protein bounded-testosterone was blocked by a confidential commercial blocking buffer provided in the kit. In addition, low-affinity testosterone antibody was adopted to ensure that the short-term binding with free testosterone did not disrupt the original testosterone–protein bound balance in body fluids. The microplate was coated with a monoclonal antibody against free testosterone, which can only recognize the specific epitope of free testosterone (this epitope is masked by SHBG/Albumin in the bound state). After adding the sample, endogenous free testosterone competed with the enzyme-labeled testosterone analog (the competitor) for the binding sites of the antibody. However, bound testosterone with proteins could not participate in the competition due to steric hindrance, and was directly washed away.

### 4.9. Vector Construction and Cell Transfection

Four DNA sequences, *07Rik*-full length+Flag, *07Rik*-5′UTR+CDS^WT^+Flag, *07Rik*-5′UTR+CDS^Mu^+Flag, and *07Rik*-CDS+Flag (Supplemental Figure S3), were designed and synthesized by GENEWIZ (Suzhou, China). The synthesized sequences were cloned into the pUC-GW-Amp plasmid individually. The plasmid with the target sequence was cut by a pair of restriction enzymes, SrfI (R0629S) and SacII (R0157S) (New England Biolabs, Ipswich, MA, USA), and was then ligated to the purified Flag-MCS-IRES-GFP-pcDNA3.1(-)-(Amp+) plasmid and transformed into *E. coli.DH5α*. After transformation, it was screened in Luria–Bertani (LB) medium with 1% (*w/v*) Tryptone, 0.5% (*w/v*) yeast extract and 1% (*w/v*) NaCl containing Ampicillin (Beyotime, Shanghai, China) resistance, and single colonies were selected after 16 h. The culture was expanded in liquid LB medium on a 37 °C shaker, and the strains with the right recombined plasmid were selected by Sanger sequencing. The plasmids were extracted with a commercial kit (AP-MN-P-250, Axygen, Union City, CA, USA) to transfect 293T cells. The cells were cultured in DMEM medium (12800017, Gibco, San Francisco, CA, USA) supplemented with 10% fetal bovine serum (RY-F22-05, Royacel, Lanzhou, China) and 1% Penicillin–Streptomycin–Gentamicin solution (Solarbio, Beijng, China) at 37 °C in a 5% CO_2_ incubator. 293T cells were planted in a 6-well plate (Nest, Wuxi, China) to about 70–80% density and then 5 µg of plasmid DNA was transfected into the cells according to Xfect™ Transfection Reagent (631317, Takara, Dalian, China). Transfection efficiency was indicated by the detection of cells with GFP fluorescent protein after 48 h. Finally, the putative *07Rik*-encoded protein was detected by Western blot or immunofluorescence, as described below.

### 4.10. Western Blot

Protein lysate was extracted from mouse testicular tissue or 293T cells with the Radio-Immunoprecipitation Assay (RIPA, Beyotime, Shanghai, China) buffer, and then the protein concentration was determined using a BCA kit (Beyotime). Equal amounts of proteins were separated with 12% SDS-PAGE gels and transferred to a PVDF membrane (Sigma, St. Louis, MO, USA) at 280 mA for 90–120 min. After blocking with 3% BSA at room temperature, the PVDF membranes loaded with protein were incubated overnight at 4 °C with the following primary antibodies: anti-Flag (F1804, Sigma), anti-3β-HSD (sc-100466, Santa Cruz Biotechnology, Dallas, TX, USA), anti-GATA4 (36966S, Cell Signaling Technology, Boston, MA, USA), anti-GAPDH (60004-1-Ig, Proteintech, Wuhan, China) and anti-Tubulin (11224-1-AP, Proteintech). The next day, membranes were washed three times with Tris Buffered Saline with Tween 20 and probed with anti-mouse or anti-rabbit IgG (H+L) (DyLight 680 Conjugate) (5366 for anti-rabbit, 5470 for anti-mouse, Cell Signaling Technology) for 1–2 h at room temperature. Western blot bands were visualized using an Amersham Typhoon system (Cytiva, Wilmington, DE, USA) and quantified using ImageJ (v1.52a, NIH, Bethesda, MD, USA) software.

### 4.11. Immunofluorescence and Immunohistochemistry

The 5 µm paraffin sections from mouse testes or fixed 293T cells were used for immunofluorescence or immunohistochemistry. For immunofluorescence, after deparaffinization and rehydration, the testicle sections were boiled in 10 mM sodium citrate buffer (pH 6.0) for 15 min and washed in phosphate buffered saline with 0.1% Triton X-100 (PBST). The samples were then blocked with the blocking buffer for 60 min and incubated with primary antibodies overnight at 4 °C. The following primary antibodies were used: anti-Flag, anti-3β-HSD, anti-GATA4 and anti–SHBG (bs-2410R, Bioss, Beijing, China). After washing the samples 3 times with PBST, Cy3-conjugated secondary antibodies (Servicebio, Wuhan, China) were used for visualization. For immunohistochemistry, the procedures from sampling to blocking were the same as those used in immunofluorescence. The blocked sections were washed with PBST three times, and we then inactivated the endogenous peroxidases with 3% H_2_O_2_ for 15 min. The primary antibody SHBG (PS05256, Abmart, Shanghai, China) and secondary antibody horseradish peroxidase conjugated goat anti-rabbit IgG(H+L) were successively incubated on the sections. Finally, the cleaned sample was used for visualization with diaminobenzidine and then staining with hematoxylin (Solarbio, Beijing, China). All samples were captured under a microscope (Axio Imager M2, Zeiss, Oberkochen, Germany).

### 4.12. Flow Cytometry

Spermatogenic cells were sorted with Hochest33342 (Beyotime, Shanghai, China) by flow cytometry, according to the processes of our previous study [35]. In brief, about 100 mg of testes were digested with HBSS solution (Hanks Balanced Salt Solution, 1.26 mM CaCl_2_, 0.49 mM MgCl_2_, 0.41 mM MgSO_4_, 5.33 mM KCl, 0.44 mM KH_2_PO_4_, 4.17 mM NaHCO_3_, 137.93 mM NaCl, 0.34 mM NaHPO_4_, 5.56 mM D-Glu, 20 mM Hepes) containing 25 U/mL Collagenase type II (07419, STEMCELL, Vancouver, BC, Canada) and 0.2 U/mL Dispase (Yuanye, Shanghai, China), and incubated for 30 min in a 37 °C incubator. Digestion was terminated by the addition of 10% FBS and the suspension was filtered through a 70 µm cell sieve (Biosharp, Hefei, China). After the cells were washed twice with HHBS by centrifugation at 900× *g* for 5 min, they were stained with Hoechst 33,342 (10 µg/mL) and incubated for 20 min in a light-proof environment. Finally, propidium iodide (10 µg/mL) and DNase I (5 U/µL) were added to the samples for sorting the various spermatogenic cells by flow cytometry (FACS Aria3, BD Bioscience, San Jose, CA, USA).

### 4.13. Statistical Analysis

All statistical analyses were performed using GraphPad Prism (v8.0.2.263) software, and considered at least three sets of independent replicates for one sample statistic. The results are given as the means ± SEM. Besides this, the differences between each group were compared using a non-paired “Student-t” test or multiple comparison with the Tukey–Kramer test. *p* < 0.05 on both sides was considered statistically significant, and *p* < 0.01 was highly statistically significant.

## Data Availability

All relevant data are given within the manuscript.

## Supplementary Materials

The following supporting information can be downloaded at: https://www.mdpi.com/article/10.3390/ijms26125801/s1.
