# Supplementary material for: lncRNA 1700009J07Rik Impaired Male Fertility by Interfering with Sexual Behaviors in Mice
Source: Int J Mol Sci. 2025 Jun 17;26(12):5801. doi: 10.3390/ijms26125801 (PMC12192799; doi:10.3390/ijms26125801)
Supplement: Supplementary file 1 [file ijms-26-05801-s001.zip › Figures S1-S6.pdf]

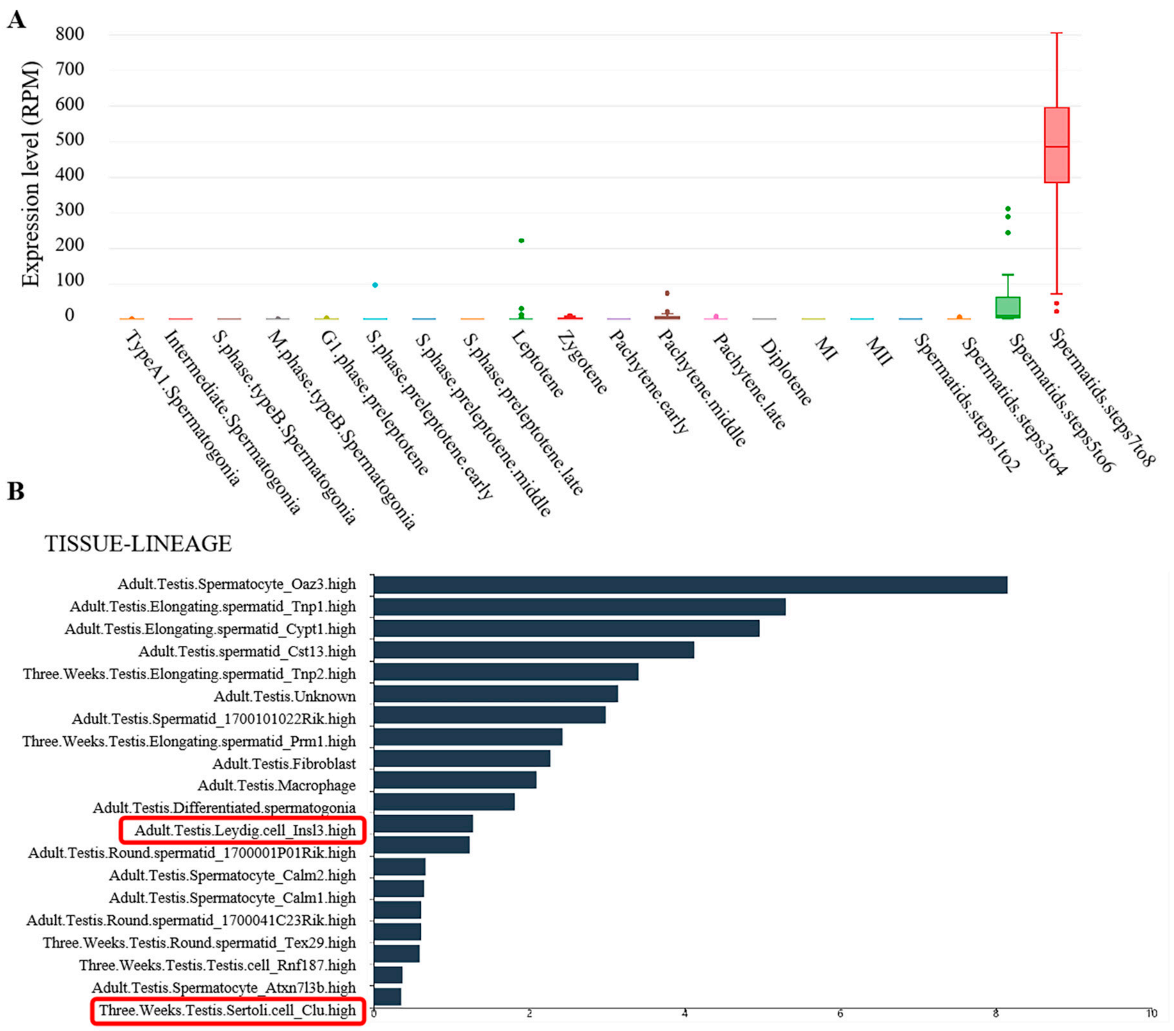

Supplemental Figure S1. 1*700009J07Rik (07Rik)* expression profiles in mouse testes. **(A)** GametesOmics single-cell transcriptome database (http://gametesomics.cn) used to investigate the expression levels of *07Rik* in spermatogenic cells. **(B)** The *07Rik* distribution in mouse testes in the Mouse Cell Atlas single-cell transcriptome database (https://bis.zju.edu.cn/MCA/index.html).

A

CPAT

Coding Potential Assessment Tool

Result for species name : mm9 with job ID :1616049897							
Data ID	Sequence Name	RNA Size	ORF Size	Ficket Score	Hexamer Score	Coding Probability	Coding Label
0	NR_015547.1	914	261	1.1619	0.415236496564	0.57410856073628	yes

B

Mark subset... Marked: 0 Download marked set as Protein FASTA

Label	Strand	Frame	Start	Stop	Length (nt | aa)
ORF4	+	3	240	500	261 | 86
ORF11	-	3	213	>1	213 | 70
ORF9	-	2	439	284	156 | 51
ORF1	+	1	424	564	141 | 46
ORF2	+	2	497	616	120 | 39
ORF7	-	2	835	725	111 | 36
ORF3	+	2	686	793	108 | 35
ORF10	-	3	738	634	105 | 34
ORF5	+	3	627	728	102 | 33
[illegible]	[illegible]	[illegible]	[illegible]	[illegible]	[illegible]

C

ORF	>lcl|ORF4
Sequence	MTTVGKGRKISVGHDHKTTVTKGHR ISLGNDHRTIVTNGVRTTVGNDHRNK VTKGHMTSVGNDHRTIVTDGLRTTV GNDYMAIVPR

Supplemental Figure S2. Coding capability assessment of *1700009J07Rik (07Rik)*. **(A)** The Coding Capability Assessment Tool (CPAT) (http://lilab.research.bcm.edu/index.php) was used to predict *07Rik*'s coding capability. **(B)** The potential open reading frames (ORF) of *07Rik*. ORFs in the *07Rik* cDNA sequence were searched by the ORF Finder (https://www.ncbi.nlm.nih.gov/orffinder/). **(C)** The supposed amino acid sequence encoded by the longest ORF of *07Rik* with an initiation codon at ATG.

A

07Rik-FL cloning vector sequence:

AGAGCAGAAGGCCCACAGGGACTCCTGGCTGCAGT
GCTGACCGAGATGGCCCCTCCCTAAAGGAGCCTGT
CTCTGAATACTTCTAGCTCCTAGAACCACTGACAGC
CTCTGACTTATGATCTCACCCCTCATTCCAAGGCCA
GCCGCTGTGGCTGTATCTCTTTTCCTCCAGAGCCTCT
GAGTCTCTCTTCTCAACCTGCTCCAGTTATCCATGA
CAACCTCCAGTCCTTTTCTTGGCAATGACCACAGTC
GGTAAGGGCCGCAAGATCTCGGTCGGCCATGATCA
CAAGACCACGGTCACCAAGGGCCACAGGATCTCAC
TTGGCAATGACCACAGGACCATAGTCACCAACGGC
GTCAGGACTACGGTTGGCAATGACCACAGGAACAA
GGTCACCAAGGGCCACATGACCTCAGTCGGCAATG
ACCACAGGACCATTGTCACCGATGGCCTCAGGACC
ACAGTTGGCAATGACTACATGGCCATTGTCCCCCGA
TGACTACAGGACCACAGTTGGCAATGGCTACAGGA
TCACGGTCTACAATAACAACAGTCAACAATGACCA
CAGAACCACAGTCAGCAGTGGAAACCTGACGTCCT
TTTCTGCTATGTAAAGAAGCAAGCATGGATTCTAGC
GTTGAGGCCCAACTCCCAAGTGACAGTGTCCAGGC
CAGCCATCAGAGATGCAGATTCCAGCCCAAGCTGC
CAGAAGCCAGCCACCACTAAGCAAGCACATCGCTT
CTTCTGAACTTCAACACCCTTCTCTGAAGCAGACCA
ACCAACCACTGTAAGAACCAACTAAAATGCCCTTG
AAAGCAAGCTGGTCCTGTCATGTGGAGGCTGAGGT
ACCCTCCCAGTGGCCCATGTCCTGAGTAAAGATAGT
GTCCTAAGGGCTGCTGCTAGCCCAATGCTATGGATT
ACAAGGATGACGACGATAAG

B

07Rik-5'UTR+CDS^WT cloning vector sequence:

AGAGCAGAAGGCCCACAGGGACTCCTGGCTGCAGTGCTGACCGAGATGGCCCCTC
CCTAAAGGAGCCTGTCTCTGAATACTTCTAGCTCCTAGAACCACTGACAGCCTCTG
ACTTATGATCTCACCCCTCATTCCAAGGCCAGCCGCTGTGGCTGTATCTCTTTTCCT
CCAGAGCCTCTGAGTCTCTCTTCTCAACCTGCTCCAGTTATCCATGACAACCTCCA
GTCCTTTTCTTGGCAATGACCACAGTCGGTAAGGGCCGCAAGATCTCGGTCGGCC
ATGATCACAAGACCACGGTCACCAAGGGCCACAGGATCTCACTTGGCAATGACCA
CAGGACCATAGTCACCAACGGCGTCAGGACTACGGTTGGCAATGACCACAGGAA
CAAGGTCACCAAGGGCCACATGACCTCAGTCGGCAATGACCACAGGACCATTGTC
ACCGATGGCCTCAGGACCACAGTTGGCAATGACTACATGGCCATTGTCCCCCGAA
TGGATTACAAGGATGACGACGATAAG

C

07Rik-5'UTR+CDS^Mu cloning vector sequence:

AGAGCAGAAGGCCCACAGGGACTCCTGGCTGCAGTGCTGACCGAGATGGCCCCTC
CCTAAAGGAGCCTGTCTCTGAATACTTCTAGCTCCTAGAACCACTGACAGCCTCTG
ACTTATGATCTCACCCCTCATTCCAAGGCCAGCCGCTGTGGCTGTATCTCTTTTCCT
CCAGAGCCTCTGAGTCTCTCTTCTCAACCTGCTCCAGTTATCCATGACAACCTCCA
GTCCTTTTCTTGGCAATTACCACAGTCGGTAAGGGCCGCAAGATCTCGGTCGGCCA
TGATCACAAGACCACGGTCACCAAGGGCCACAGGATCTCACTTGGCAATGACCAC
AGGACCATAGTCACCAACGGCGTCAGGACTACGGTTGGCAATGACCACAGGAACA
AGGTCACCAAGGGCCACATGACCTCAGTCGGCAATGACCACAGGACCATTGTCAC
CGATGGCCTCAGGACCACAGTTGGCAATGACTACATGGCCATTGTCCCCCGAATG
GATTACAAGGATGACGACGATAAG

D

07Rik+CDS cloning vector sequence:

ATGACCACAGTCGGTAAGGGCCGCAAGATCTCGGTCGGCCATGATCACAAGACCA
CGGTCACCAAGGGCCACAGGATCTCACTTGGCAATGACCACAGGACCATAGTCAC
CAACGGCGTCAGGACTACGGTTGGCAATGACCACAGGAACAAGGTCACCAAGGG
CCACATGACCTCAGTCGGCAATGACCACAGGACCATTGTCACCGATGGCCTCAGG
ACCACAGTTGGCAATGACTACATGGCCATTGTCCCCCGATGAATGGATTACAAGG
ATGACGACGATAAG

5'UTR CDS 3'UTR

T Mutation base Flag

Supplemental Figure S3. The constructed vector sequences with different *1700009J07Rik (07Rik)* transcript fragments. **(A)** Full-length *07Rik* sequence with Flag tag. **(B)** 5′UTR + coding sequence (CDS) of *07Rik* with Flag tag. **(C)** 5′UTR + mutated the start codon (Changed ATG to ATT) of the *07Rik* sequence with Flag tag. **(D)** *07Rik* CDS with Flag tag.

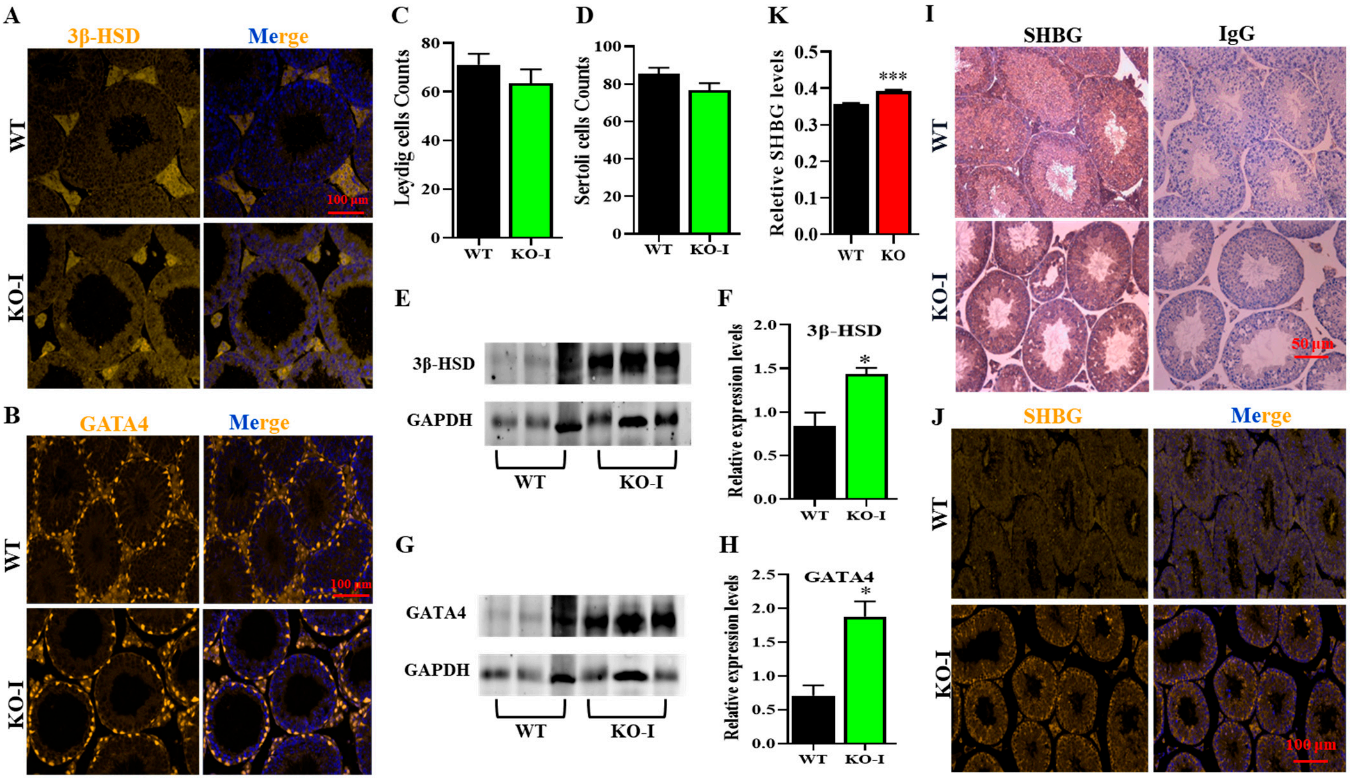

Supplemental Figure S4. Evaluation of the counts and functions of Leydig and Sertoli cells in *07Rik* knockout (KO) mice. **(A)** Immunofluorescence staining with the marker of Leydig cells 3β-HSD in the testes. **(B)** Immunofluorescence staining with the marker of Sertoli cells GATA4 in the testes. **(C)**, **(D)** Statistical analysis of the numbers of Leydig and Sertoli cells from **(A)** and **(B)** after *07Rik* KO in male mice. **(E)**, **(G)** The effect of *07Rik* deletion on 3β-HSD and GATA protein expression levels. **(F)**, **(H)** Statistical analysis of the protein levels of 3β-HSD and GATA from **(E)** and **(G)**. **(I)** Immunohistochemical staining of SHBG in the KO-I testes. **(J)** Immunofluorescence staining of SHBG in the KO-I testes. **(K)** Statistical analysis of the protein levels of SHGB from **(J)**. The expression levels of the SHBG protein in testicular sections or the protein lysate were detected by immunofluorescence or immunohistochemistry with an SHBG antibody. IgG acted as a negative control. GAPDH was used as a loading control to normalize the protein expression levels. All data are means ± SEM (n = 3). *P* values were calculated using the two-tailed Student's t test. * *P* < 0.05, *** *P* < 0.001, compared with the wild-type (WT) group. SHGB: sex hormone-binding globulin.

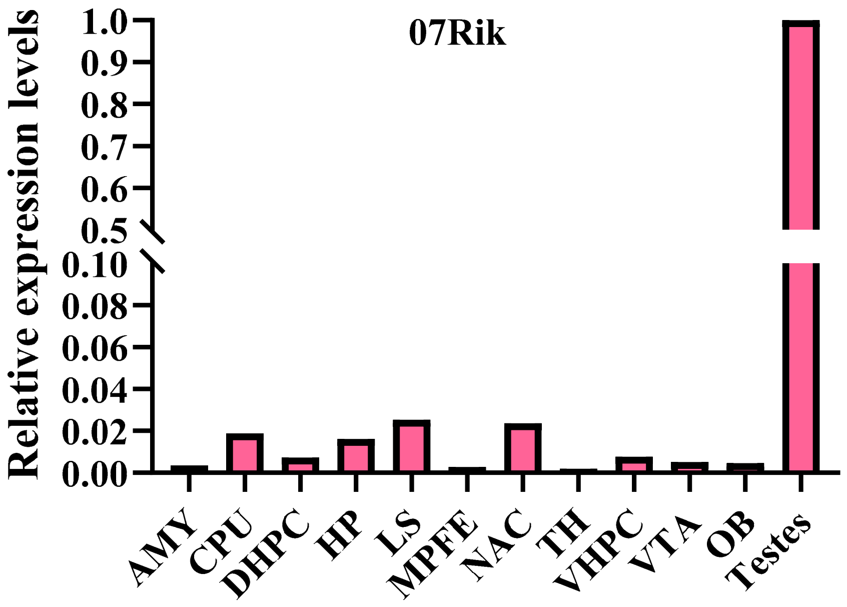

Supplemental Figure S5. Transcript expression levels of *1700009J07Rik (07Rik)* in the different brain areas. Total RNA was isolated from various brain tissues, and *07Rik* expression levels were tested by qPCR as described in the Material and Methods Section. AMY: amygdaloid nucleus. CPU: dorsal striatum. DHPC: dorsal hippocampus. HP: hypothalamus. LS: lateral septal nuclei. MPFC: prefrontal cortex. NAC: nucleus accumbens. TH: thalamus. VHPC: ventral hippocampus. VTA: Volume of Tissue Activation. OB: olfactory bulb.

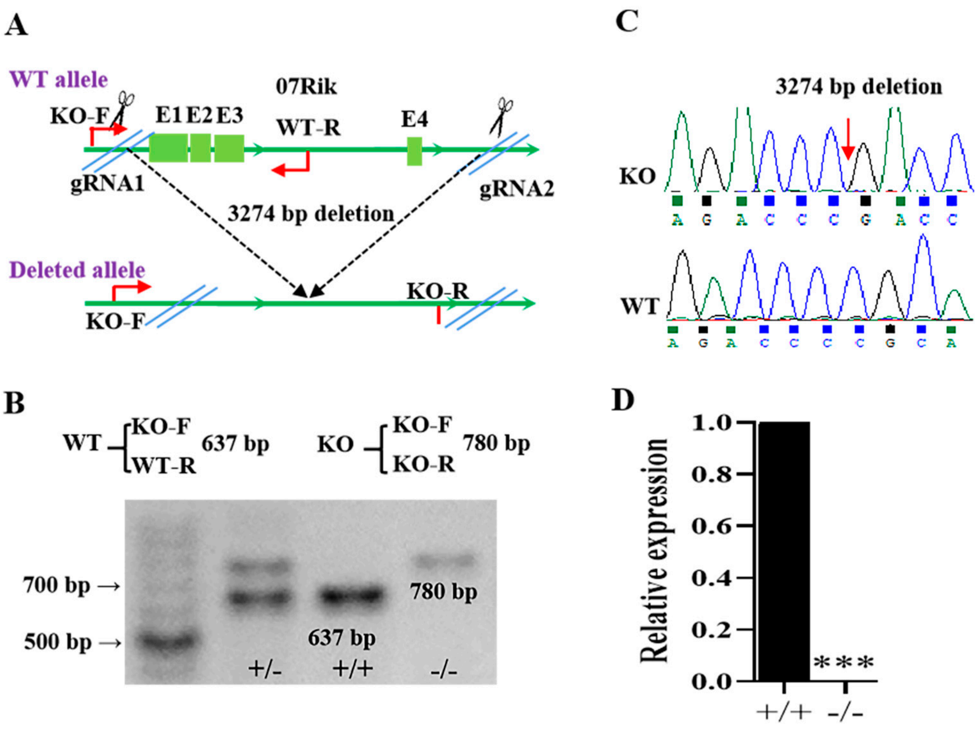

Supplemental Figure S6. Construction and validation of *1700009J07Rik* (*07Rik*) knockout (KO) mice. **(A)** A schematic illustration of the Clustered Regularly Interspaced Short Palindromic Repeat (CRISPR)-Cas9-generated *07Rik* KO mice. A pair of guide RNAs (gRNA1 and gRNA2) was employed to target and excise a 3274 bp genomic DNA fragment of *07Rik*. **(B)** Genotypic identification of KO mice. The PCR analysis of mouse tail DNA revealed distinct bands of 637 bp and 780 bp in KO and wild-type (WT) mice, respectively. **(C)** Sanger sequencing was conducted using PCR products to prove the successful KO of the *07Rik* DNA fragment. **(D)** Expression levels of the *07Rik* transcript in KO and WT mice. Total RNA was isolated from the testes using a MiniBEST Universal RNA Extraction kit and the *07Rik* expression levels were detected by qPCR. *Gapdh* was used as a loading control to normalize the gene expression levels. The data are means ± SEM (n = 3). P values were calculated using the two-tailed Student's t test. ***P < 0.001, compared with the group of WT.
